# Editorial: Dietary bioactive compounds on chronic diseases chemoprevention: from molecular mechanism to clinical application and beyond

**DOI:** 10.3389/fimmu.2024.1453272

**Published:** 2024-07-08

**Authors:** Pengcheng Huang, De-Xing Hou, Qiang Wang, Hong Chen, Zhaoyun Ding, Si Qin, Wuquan Deng

**Affiliations:** ^1^ Department of Endocrinology and Metabolism, School of Medicine, Chongqing University Central Hospital, Chongqing Emergency Medical Centre, Chongqing University, Chongqing, China; ^2^ Kagoshima University, Kagoshima, Kagoshima, Japan; ^3^ Wuhan University of Science and Technology, Wuhan, Hubei, China; ^4^ Sichuan Agricultural University, Ya'an, Sichuan, China; ^5^ National University of Defense Technology, Changsha, Hunan, China; ^6^ Hunan Agricultural University, Changsha, Hunan, China

**Keywords:** bioactive compounds, inflammation, mechanisms of action, lifestyle interventions, gut microbes

Bioactive substances are trace or small amounts of substances from living organisms, and they have a significant impact on various biological processes. These substances, when ingested through dietary sources, play a crucial role in maintaining normal bodily functions and improving human health. In this editorial, we explore the multifaceted roles of dietary active compounds, the molecular mechanisms that influence chronic diseases, and highlight their impact on human health.

In this Research Topic, two detailed reviews described the functions of bioactive substances in promoting human metabolism and improving human health. El-Saadony et al. summarized the biological functions and mechanisms of garlic and its main bioactive components in disease prevention and treatment. Garlic contains biological sulfur compounds, including allicin, which are thought to be the main pharmacologically active ingredients in the matrix because of their strong antioxidant, antibacterial, and anticancer effects. One important aspect of garlic’s biological components’ ability to promote health is their absorption in the intestines. The authors reviewed the role of garlic in the liver and kidneys, the digestive system, the cardiovascular system and the nervous system, described its antibacterial, anti-inflammatory, antihypertensive, antidiabetic and anti-obesity properties, and analyzed its potential as an antioxidant and anticancer agent that we can have a comprehensive understanding of the health-promoting properties of garlic.

Dietary bioactive signaling influences the gut microbiota, immune system and pain responses via the gut-immune-bone- axis. Basak et al. highlighted recent advances in mechanistic evidence that bioactivity had been shown to reduce the pathophysiology of osteoarthritis. Toll-like Receptor 4 (TLR4), a key protein regulating intestinal permeability and involved in acute and chronic inflammation, has been an important link between the gut microbiota and immune responses generated by the immune system. Certain specific microbial metabolites interact directly with exogenous receptors via TLR4 signaling to modulate gut permeability causing an inflammatory response and affecting bone remodeling, allowing an imbalance between osteoclastogenesis (bone resorption) and osteoblastogenesis (bone formation). Dietary habits are largely responsible for maintaining the balance of the intestinal internal environment. Bioactive substances, which have been shown to have multiple health benefits, modulate microbiota composition and influence the immune system in the presence of inflammatory disorders. Regarding osteoarthritis, the innate immune system plays a crucial role in inducing an inflammatory response during disease progression. This study highlighted the importance of bioactive compounds in the fight against osteoarthritis, exploring their exact molecular mechanisms and therapeutic targets, which will contribute to the early treatment of osteoarthritis, the prevention of disability and the improvement of patients’ quality of life.

The clinical trial contributed by Xu et al. is an exploration of the effect of lifestyle intervention on pregnancy outcomes in high-risk groups of gestational diabetes mellitus (GDM). Twenty-three participants were randomly assigned to a control group consisting of routine pregnancy check-ups, whereas 251 women at high risk of GDM and 128 randomly assigned to lifestyle treatments (dietary guidance, health education, and weight management) were included in the study. This intervention prevented metabolic abnormalities that may occur due to inadequate nutritional intake during pregnancy and ultimately reduced the incidence of gestational diabetes mellitus and hyperemesis gravidarum. This study highlights the importance of early screening and intervention for high-risk pregnant women.

Li et al. identified Lactobacillus plantarum can alleviate glucocorticoid-induced osteoporosis. This study investigated the effect of probiotic Lactobacillus plantarum on bone health and its mechanism in rats with glucocorticoid dexamethasone-induced osteoporosis, using sodium alemphosphate, a drug used in the treatment of osteoporosis, as a reference. The authors observed bone microjunctions and analyzed gut microbiota and serum metabolites in rats and found that bone microstructural parameters were significantly restored in the Lactobacillus plantarum-treated group, with an increase in bone density, an increase in the number and thickness of bone trabeculae, a significant increase in the abundance of beneficial bacteria and a significant decrease in the abundance of harmful bacteria, and an even greater abundance of metabolites, which promoted the formation of osteoblasts. This study suggests that Lactobacillus plantarum is a potential candidate for the treatment of periodontitis.

In summary, the results of this Research Topic demonstrate how the intake of dietary active compounds, especially phytochemicals, can reduce the risk of chronic disease by reducing oxidative stress, activating inflammatory pathways and remodeling the gut flora. With the advent of the era of nutrigenomics, we anticipate that we can conduct further molecular mechanism studies targeting the redox-related signaling pathways of chronic diseases and the role of dietary active compounds in the chemoprevention of chronic diseases, as well as the interactions between these pathways and the gut flora *in vivo* and *in vitro*, which can pave the way for further deepening of our research on dietary active compounds and help us to understand the overall meaning of life and promote us to lead a healthier life.

## Author contributions

PH: Writing – original draft, Writing – review & editing. WD: Writing – original draft, Writing – review & editing. D-XH: Writing – original draft, Writing – review & editing. QW: Writing – original draft, Writing – review & editing. HC: Writing – original draft, Writing – review & editing. ZD: Writing – original draft, Writing – review & editing. SQ: Writing – original draft, Writing – review & editing.

